# The endoplasmic reticulum stress/autophagy pathway is involved in cholesterol-induced pancreatic β-cell injury

**DOI:** 10.1038/srep44746

**Published:** 2017-03-15

**Authors:** Fei-Juan Kong, Jia-Hua Wu, Shui-Ya Sun, Jia-Qiang Zhou

**Affiliations:** 1 Department of Endocrinology, Sir Run Run Shaw Hospital, School of Medicine, Zhejiang University, Hangzhou, China

## Abstract

Lipotoxicity has been implicated in pancreatic β-cell dysfunction in type 2 diabetes, but the exact mechanisms remain unknown. The current study explored the role of the endoplasmic reticulum (ER) stress pathway in cholesterol-induced lipotoxicity. Two different insulinoma cell lines were treated with cholesterol with or without inhibitors. ER stress-associated proteins glucose-regulated protein (GRP) 78, activating transcription factor (ATF) 4 and C/EBP homologous protein (CHOP), as was phosphorylation of eukaryotic initiation factor (EIF) 2α, were all up-regulated by cholesterol. Cholesterol also up-regulated microtubule-associated protein 1 light chain 3 (LC3)-II and stimulated the formation of autophagic vacuoles and LC3-II aggregates. Cholesterol-induced autophagy and cell injuries were suppressed by pretreatment with the ER stress inhibitor 4-phenylbutyrate (4-PBA). Pretreatment with autophagy inhibitors E-64d/pepstatin A increased ER stress-induced cell injuries as indicated by increased cell apoptosis and decreased insulin secretion. These results suggest that cholesterol treatment induces apoptosis and dysfunction of β-cells, and enhances autophagy through activation of the ER stress pathway. More importantly, autophagy induced by cholesterol may protect β-cells against ER stress-associated cell damages.

The past two decades have witnessed the increasing worldwide prevalence of diabetes mellitus (DM), especially type 2 diabetes mellitus (T2DM). T2DM is a progressive metabolic disorder characterized by insulin resistance and dysfunctional pancreatic β-cells. Evidence indicates that cell dysfunction induced by lipid accumulation or lipotoxicity in pancreatic β-cells may contribute to the pathogenesis of T2DM^1^,^2^,^3^,^4^. Lipid accumulation or lipotoxicity is typified by high levels of free fatty acids (FFAs), low-density lipoprotein (LDL), triglycerides, and cholesterol^5^. Most prior studies have mainly focused on free fatty acids that induce β-cell death in cultured cells and isolated islets^1^,^4^. Recent studies have indicated that cholesterol metabolism is closely related to β-cell function. Normally, there is a dynamic balance in β-cell cholesterol content by a series of receptors and transporters that control its influx and efflux. The disorder of cholesterol metabolism will interfere with the glucose metabolism and insulin secretion^2^, which could eventually lead to cell apoptosis^1^,^4^,^6^,^7^. However, the underlying mechanisms are complicated and remain poorly understood.

The endoplasmic reticulum (ER) is a highly dynamic organelle responsible for the synthesis, folding and assembly of almost all secreted and transmembrane proteins, as well as intracellular calcium homeostasis. Extracellular or intracellular stimuli that perturb ER function lead to the accumulation and aggregation of unfolded and/or misfolded proteins in the ER, causing ER stress and subsequent activation of stress-related signaling pathways. Moderate ER stress serves as a protective mechanism by activating the unfolded protein response (UPR) to preserve ER homeostasis^8^. However, if the adaptive responses are not sufficient to relieve ER stress, apoptosis is initiated, and cells enter programmed death. ER stress and/or ER stress-induced apoptosis is increasingly recognized as an important mechanism in the development of DM, not only for β-cell loss but also for insulin resistance^9^,^10^. Accumulating evidence suggests that ER stress-induced apoptosis may be an important mode of β-cell loss^10^. Recently, ER stress has been proposed to be associated with lipotoxicity-induced β-cell apoptosis^11^,^12^. In the current study, we hypothesized that ER stress-mediated apoptotic pathways play a role in the mechanisms of cholesterol-induced β-cell apoptosis.

Macroautophagy (hereafter referred to as autophagy) is a physiologically conserved process involving the degradation of cytoplasmic material and maintenance of cellular homeostasis by eliminating damaged organelles and aggregated proteins. The double-membrane autophagosome or autophagic vacuole forms and sequesters cytoplasmic material, during which microtubule-associated protein 1 light chain 3 (LC3, a mammalian ortholog of yeast Atg8) changes from LC3-I to LC3-II, followed by translocation to the autophagosome membrane^13^. The autophagosome then fuses with the lysosome to form an autolysosome, in which the sequestered cargo is degraded by lysosome hydrolytic enzymes. Recently, autophagy has attracted increasing attention due to its role in regulating β-cell function^14^ and cell death^15^. In addition, autophagy plays an important role in maintaining the structural and functional integrity of the ER^16^, which is crucial to the survival and function of β-cells^17^. ER stress and autophagy are both associated with lipotoxicity in β-cells, but the exact nature of the link between ER stress and autophagy remains unclear.

In the current study, we explored the role of the ER stress pathway in cholesterol-induced autophagy and apoptosis in pancreatic β-cells. In addition, we investigated the influence of autophagy on ER stress-induced pancreatic β-cell injuries.

## Results

### Cholesterol treatment induces apoptosis in pancreatic β-cells

**Figure 1:**
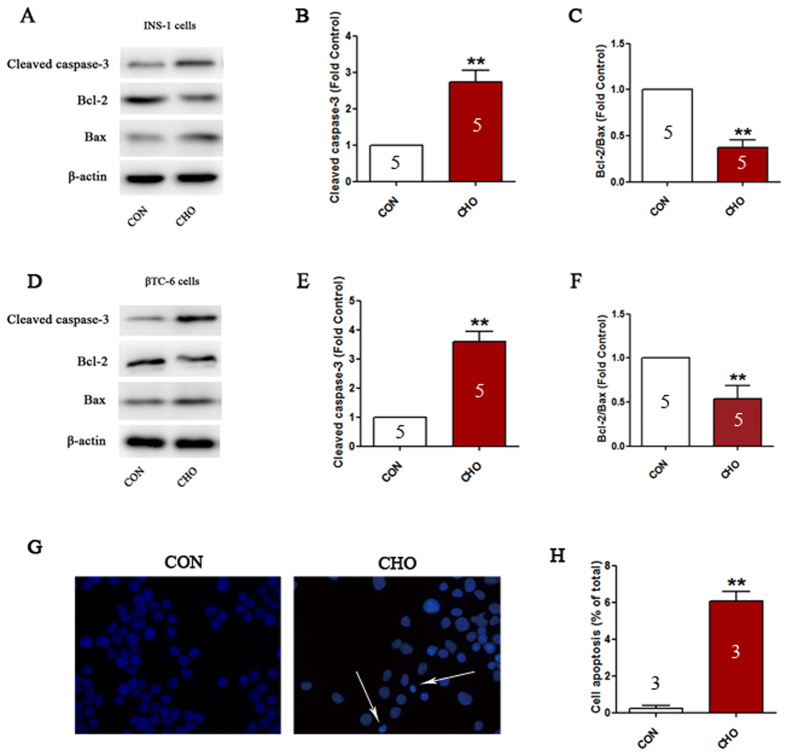
Cholesterol treatment induces apoptosis in pancreatic β-cells. Expression of cleaved caspase-3, bcl-2, and bax in INS-1 cells (**A**–**C**) and βTC-6 cells (**D**–**F**) determined by western blotting. Apoptosis in INS-1 cells was detected using DAPI staining (**G**,**H**). Arrows indicate apoptotic cell nuclei. Data are expressed as the mean ± SD values of three to five independent experiments. Statistical significance was assessed using the Student’s *t* test. *P < 0.05, **P < 0.01 compared to CON. CON = controls; CHO = cholesterol.

Cholesterol treatment led to an increase in the expression of apoptosis-related proteins, as indicated by an increase in the expression of cleaved caspase-3 and a decrease in the ratio of bcl-2 and bax (P < 0.01, Fig. 1A–F). In addition, immunofluorescence analysis indicated that cholesterol treatment resulted in significant cell apoptosis (nuclear shrinkage or fragmentation) in 6.1 ± 0.49% of cells (Fig. 1G,H).

### Cholesterol treatment activates ER stress in pancreatic β-cells

**Figure 2:**
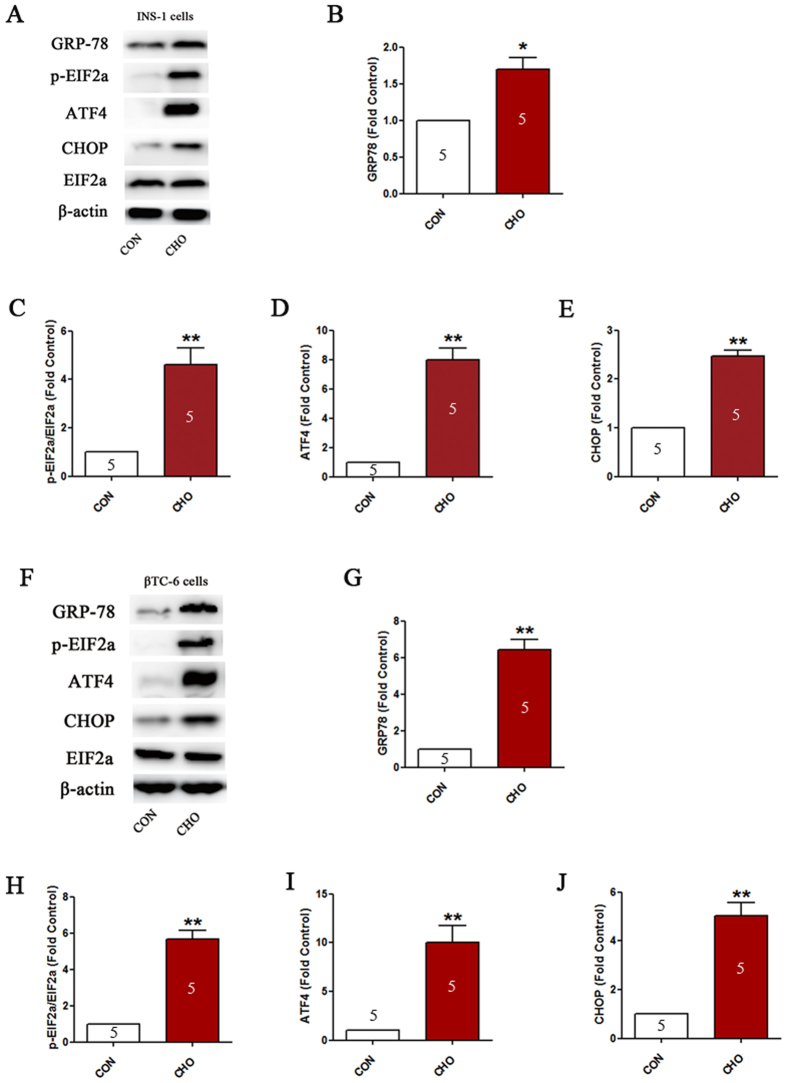
Cholesterol treatment activates ER stress in pancreatic β-cells. Expression of GRP78, p-EIF2α, ATF4 and CHOP in INS-1 cells (**A**–**E**) and βTC-6 cells (**F**–**J**) determined by western blotting. Data are expressed as the mean ± SD values of three to five independent experiments. Statistical significance was assessed using the Student’s *t* test. *P < 0.05, **P < 0.01 compared to CON. CON = controls; CHO = cholesterol.

To determine whether ER stress was triggered by cholesterol application in β-cells, we detected changes in ER stress pathway markers. The results revealed that the expression of ER stress-associated proteins GRP78 (P < 0.05, Fig. 2A,B,F and G) and ATF4 (P < 0.01, Fig. 2D and I), as well as phosphorylation of EIF2α (P < 0.01, Fig. 2C and H), were up-regulated following cholesterol treatment. As one of the components of the ER stress-mediated apoptotic pathway, the CHOP protein level of the cholesterol group was significantly increased in contrast to that of the control group (P < 0.01, Fig. 2E and J).

### Cholesterol treatment stimulates autophagy in pancreatic β-cells

**Figure 3:**
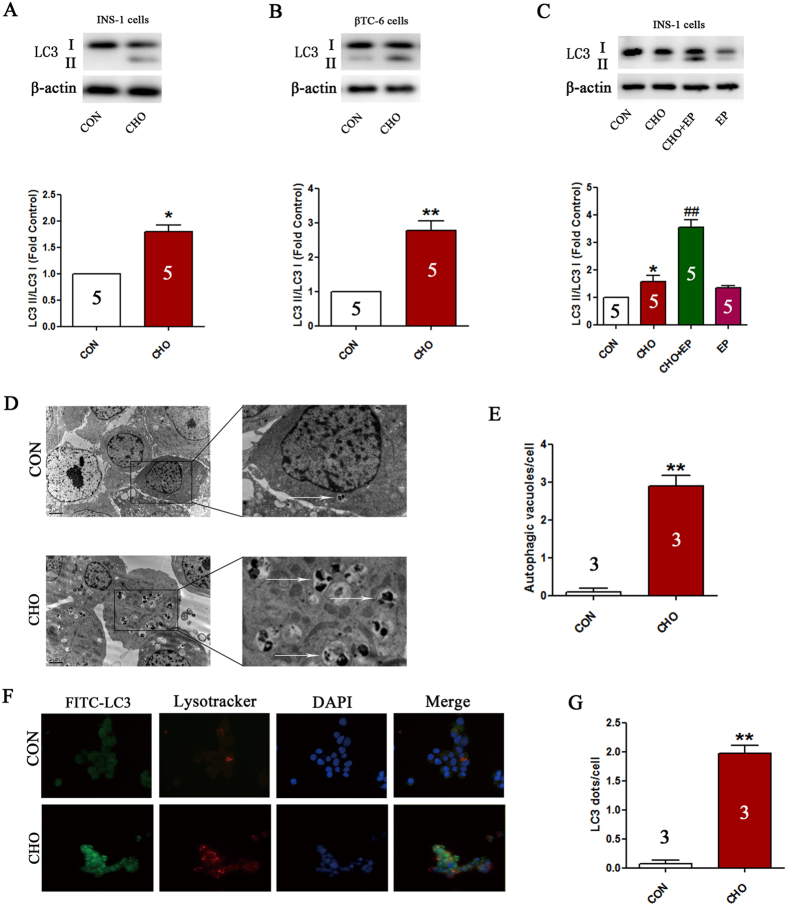
Cholesterol treatment stimulates autophagy in pancreatic β-cells. Expression of LC3-II in INS-1 and βTC-6 cells determined by western blotting (**A**–**C**). Formation of autophagic vacuoles in INS-1 cells was estimated using TEM (**D**,**E**). Arrows indicate autophagic vacuoles. Formation of LC3-II point-like aggregates in INS-1 cells evaluated using immunofluorescence (**F**,**G**). Data are expressed as the mean ± SD values of three to five independent experiments. Statistical significance was analyzed using the Student’s *t* test and one-way ANOVA. Statistical significance was determined by *P < 0.05, **P < 0.01 compared to CON, ^#^*P* < 0.05, ^##^*P* < 0.01 compared to CHO. CON = controls; CHO = cholesterol; EP = E64d + pepstatin A.

The results of western blotting revealed that cholesterol treatment enhanced the levels of the autophagic marker LC3-II compared with that of the control group (P < 0.05, Fig. 3A,B). To assess whether the increased LC3-II levels were due to enhancement of autophagic flux or blockage of autophagic degradation, E64d combined with pepstatin A, which inhibits lysosomal enzymes and interferes with autolysosomal digestion, was used in our study. In addition, the trend mentioned above was even more obvious in the presence of the lysosomal protease inhibitor E64d/pepstatin A (P < 0.01, Fig. 3C), indicating that increased LC3-II levels were probably due to increased autophagic activity. Additionally, transmission electron microscopy (TEM) and immunofluorescence analysis further confirmed the formation of autophagic vacuoles and LC3-II point-like aggregates (bright green fluorescent spots) following cholesterol treatment (P < 0.01, Fig. 3D–G).

### Inhibition of ER stress attenuates cholesterol-induced β-cell injuries and autophagy

**Figure 4:**
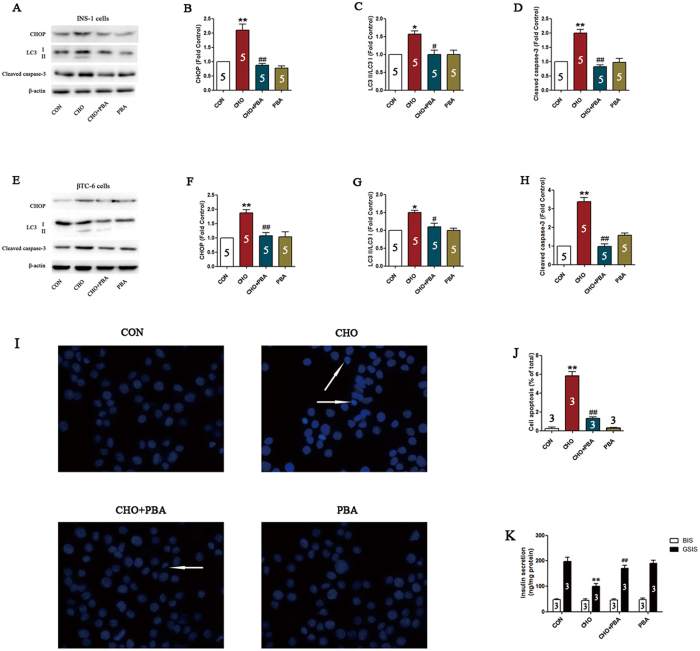
Inhibition of ER stress attenuates cholesterol-induced β-cell injury and autophagy. Expression of CHOP, LC3-II and cleaved caspase-3 in INS-1 cells (**A**–**D**) and βTC-6 cells (**E**–**H**) determined by western blotting. Apoptosis in INS-1 cells was detected using DAPI staining (**I**,**J**). Arrows indicate apoptotic cell nuclei. Insulin secretion in βTC-6 cells was evaluated using an ELISA kit and normalized against protein content (**K**). Data are expressed as the mean ± SD values of three to five independent experiments. Statistical significance was analyzed using a one-way ANOVA followed by Tukey’s multiple comparison testing. Statistical significance was determined by *P < 0.05, **P < 0.01 compared to CON, ^#^*P* < 0.05, ^##^*P* < 0.01 compared to CHO. CON = control; CHO = cholesterol; PBA = 4-phenylbutyrate.

To assess the potential involvement of the ER stress pathway in the activation of autophagy and induction of β-cell injuries, the inhibitor of ER stress 4-PBA was used, which is supposed to interact with the hydrophobic domains of misfolded proteins, thus preventing their aggregation. The results showed that pretreatment with 4-PBA reversed the up-regulation of LC3-II in response to cholesterol (P < 0.05, Fig. 4A,C,E and G). Moreover, cholesterol-induced activation of apoptosis-related proteins, such as cleaved caspase-3, and cell apoptosis, were also attenuated by 4-PBA pretreatment (P < 0.01, Fig. 4D and H–J). As a vital function of β-cells, insulin secretion was also evaluated in our study. Basal insulin secretion (BIS) did not show significant changes among the four groups, while glucose-stimulated insulin secretion (GSIS) was decreased by cholesterol treatment and reversed by 4-PBA application (P < 0.01, Fig. 4K).

### Inhibition of autophagy aggravates cell injuries caused by ER stress

**Figure 5:**
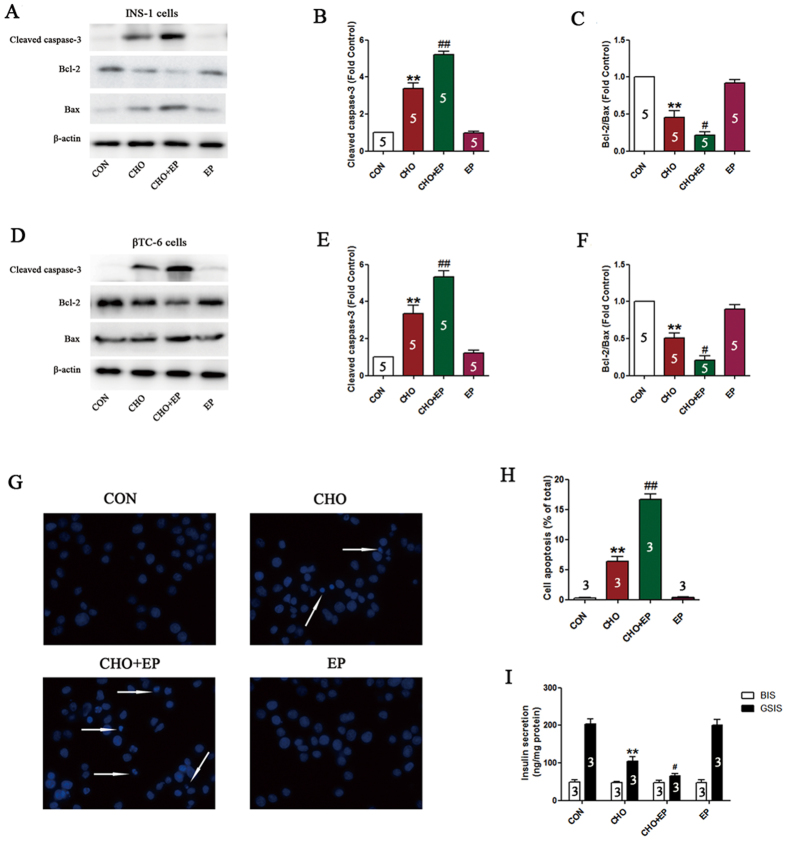
Inhibition of autophagy aggravates ER stress-induced cell injuries. Expression of cleaved caspase-3, bcl-2, and bax in INS-1 cells (**A**–**C**) and βTC-6 cells (**D**–**F**) determined by western blotting. Apoptosis in INS-1 cells was detected using DAPI staining (**G**,**H**). Arrows indicate apoptotic cell nuclei. Insulin secretion in βTC-6 cells was evaluated using an ELISA kit and normalized against protein content (**I**). Data are expressed as the mean ± SD values of three to five independent experiments. Statistical significance was analyzed using one-way ANOVA followed by Tukey’s multiple comparison testing. Statistical significance was determined by *P < 0.05, **P < 0.01 compared to CON, ^#^*P* < 0.05, ^##^*P* < 0.01 compared to CHO. CON = controls; CHO = cholesterol; EP = E64d+ pepstatin A.

The trends in increased levels of cleaved caspase-3 and decreased ratio of bcl-2 and bax induced by cholesterol were further exacerbated by cholesterol exposure following pretreatment with E64d/pepstatin A, as was cell apoptosis (P < 0.05, Fig. 5A–H), and the cholesterol-induced decrease in insulin secretion was even more pronounced (P < 0.05, Fig. 5I).

## Discussion

In the present study, we investigated the involvement of the ER stress signaling pathway in pancreatic β-cell autophagy and apoptosis induced by cholesterol. We also investigated the effect of autophagy on cholesterol-induced β-cell injury. Cholesterol stimulated autophagy and apoptosis in both INS-1 and βTC-6 pancreatic β-cell lines, which were associated with the up-regulated or activated ER stress proteins GRP78, p-EIF2α, ATF4 and CHOP. Importantly, inhibition of autophagy further aggravated cell injuries induced by cholesterol.

Diabetes is a significant global public health problem affecting more than 285 million people worldwide. In addition to increasing the economic burden on families and society, T2DM also leads to a variety of complications and an increased incidence of mortality. Given the rapidly increasing number of patients with T2DM, it is crucial to improve our understanding of the underlying molecular mechanisms to explore more appropriate treatment options. Maintaining pancreatic β-cell mass and function is essential for preventing T2DM, and β-cell apoptosis is a critical contributing factor in the development of the disease. Lipotoxicity is increasingly recognized as a leading cause of pancreatic β-cell loss. The balance of cholesterol metabolism is necessary to maintain the functions of β-cells, such as insulin secretion. When the balance is broken, it may cause cell apoptosis. In the present study, we added exogenous cholesterol to INS-1 cells and βTC-6 cells *in vitro* to raise the level of cholesterol. We found that cholesterol treatment up-regulated a number of apoptosis-related proteins. Specifically, cleaved caspase-3 expression was increased, and the bcl-2 and bax ratio was decreased, while β-cell apoptosis was elevated. Secretion of insulin is among the most important function of β-cells, and this was diminished following cholesterol exposure. Consistent with previous studies^3^,^6^,^7^, these results confirmed that cholesterol treatment caused injury to β-cells that included stimulating apoptosis and reducing insulin secretion.

The molecular mechanisms underlying the above results are poorly understood, although lipotoxicity is reported to induce ER stress and apoptosis in β-cells^12^. Insulin resistance has been found to be detected in almost all T2DM patients. Under this condition, more insulin is required to maintain the balance of glucose metabolism. Pancreatic β-cells are highly specialized for insulin production and secretion and contain highly developed ERs and a robust ER signaling system for this purpose, which makes them extremely prone to ER stress when insulin production overwhelms the protein folding capacity of the ER^18^. ER stress results from disruption to homeostasis following overstimulation, causing dissociation of the 78-kDa glucose-regulated protein (GRP78) from the three ER transmembrane effector proteins protein kinase RNA (PKR)-like ER kinase (PERK), inositol-requiring enzyme 1 (IRE1), and activating transcription factor 6 (ATF6). This stimulates the unfolded protein response (UPR), which relieves cellular dysfunction and enhances cell survival^19^.

However, a prolonged UPR can trigger ER stress-mediated apoptosis, leading to the expression of the pro-apoptotic transcription factor CHOP and ultimately β-cell apoptosis^9^. Recent studies have shown that PERK-ATF4-CHOP stress signaling is important in β-cell apoptosis^20^,^21^. PERK activation by phosphorylation leads to phosphorylation of eukaryotic initiation factor (EIF) 2α, which in turn leads to translational attenuation and induction of ATF4, resulting in the eventual activation of CHOP which up-regulates pro-apoptotic proteins and down-regulates anti-apoptotic proteins to promote apoptosis. Consistently, our studies showed that cholesterol treatment induced ER stress and the ER stress-mediated apoptosis pathway, as indicated by increased levels of GRP78, p-EIF2α, ATF4 and CHOP. Furthermore, pretreatment with the ER stress inhibitor 4-PBA attenuated the up-regulation of caspase-3 and cell apoptosis in response to cholesterol and reversed the decrease in insulin secretion. These results further confirmed that ER stress is involved in cholesterol-induced β-cell injury, and the CHOP-mediated apoptosis pathway may partially contribute to ER stress-induced cell death. However, a previous study suggested that free cholesterol loading did not affect expression levels of ER stress markers in ΜΙΝ6 cells^7^. These discrepancies could be due to methodological differences, cell species differences, pharmacological differences, or differences in concentrations or durations.

Increasing evidence suggests that autophagy is associated with lipotoxicity^12^,^22^, and our study provides new evidence to further confirm the issue. However, the potential mechanisms are still not clear. Activation of autophagy is recognized as a novel signaling pathway in response to ER stress and is reported to be mediated by the ER stress pathway. The IREI-TRAF2-JNK pathway^23^ and PERK-EIF2a signaling^24^ are reportedly involved, along with AKT-TSC-mTOR signaling^25^. Our results showed that cholesterol induced autophagy in β-cells, along with the activation of ER stress signaling. Pretreatment with the ER stress inhibitor 4-PBA reversed the up-regulation of LC3-II induced by cholesterol. The data presented in this report demonstrated that cholesterol up-regulated autophagy in β-cells, which might be associated with activation of the ER stress-induced PERK-EIF2a pathway.

**Figure 6:**
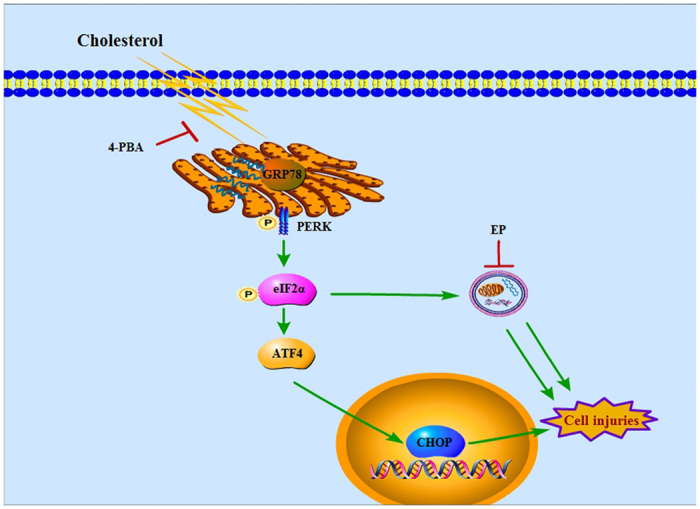
Model of the pathway of cholesterol-induced ER stress and autophagy in β-cells. Cholesterol stimulates autophagy and cell injuries in pancreatic β-cell lines, which are associated with the up-regulation or activation of ER stress proteins GRP78, p-EIF2α, ATF4 and CHOP. Inhibition of autophagy further aggravates cell injuries induced by cholesterol.

Now, it is generally accepted that autophagy promotes cell survival under some circumstances and participates in cell death in others. Autophagy has previously been shown to protect against lipotoxicity-induced β-cell injury. Induction of autophagy by rapamycin decreased palmitate-induced apoptosis in INS-1 β-cells, and inhibition of autophagy by deletion of autophagy-related Atg 5 genes, bafilomycin A1 or E64d/pepstatin A, augmented β-cell death^12^. Similarly, the current study demonstrated that cholesterol exposure following E64d/pepstatin A led to a further increase in apoptosis-related proteins and cell apoptosis and an even bigger decrease in insulin secretion than treatment with cholesterol alone. These results suggested that, on the one hand, cholesterol treatment resulted in lipotoxicity as demonstrated by enhancement in β-cell apoptosis and reduction in insulin secretion; on the other hand, it activated autophagy (as shown in Fig. 6). In addition, the stimulation of autophagy under stress conditions exhibited a protective role against lipotoxicity-induced β-cell damage, indicating that the up-regulation of the autophagy level was essential to β-cell survival, although cell injury was not completely reversed by this degree of autophagy. Interestingly, previous studies have shown that autophagy was also activated in T2DM patients^24^. Therefore, moderate up-regulation of the autophagy level may be a promising therapeutic target for this disorder. However, other evidence indicates that autophagic machinery can be recruited to kill cells via a caspase-independent autophagic cell death mechanism^26^. While the role of autophagy in the maintenance of β-cell function is beyond a doubt, the relationship between lipotoxicity and autophagy is more complicated than originally thought and may differ depending on cell type, experimental methods and assay conditions. More sophisticated studies are needed to elucidate the exact underlying mechanisms.

In conclusion, our results show that cholesterol treatment induces apoptosis and dysfunction of pancreatic β-cells and enhanced autophagy through activation of the ER stress pathway. Blocking autophagy with specific inhibitors aggravates ER stress-mediated cell injuries, suggesting that autophagy may serve to protect against stress-associated cell damage in pancreatic β-cells.

## Materials and Methods

### Materials

INS-1 rat insulinoma cells were kindly donated by Prof. Yingke Xu (Zhejiang University, Hangzhou, Zhejiang, China), and βTC-6 mouse insulinoma cells were purchased from the Cell Bank of the Shanghai Institute of Cell Biology (Chinese Academy of Sciences, Shanghai, China). Fetal bovine serum (FBS) was obtained from either Biochrom (Berlin, Germany) or GIBCO (Carlsbad, CA, USA). Dulbecco’s modified Eagle’s medium (DMEM) and RPMI 1640 medium were from GIBCO (Carlsbad, CA, USA). All chemicals, including cholesterol, 4′,6-diamidino-2-phenylindole (DAPI), 4-phenylbutyrate (4-PBA; chemical chaperone), (2S, 3S)-trans-epoxysuccinyl-L-leucylamido-3-methylbutane ethyl ester (E64d; inhibitor of lysosomal protease), and pepstatin A (inhibitor of lysosomal protease), were purchased from Sigma-Aldrich Corp. (St. Louis, MO, USA). The Lyso Tracker Red Lysosomal Probe was obtained from Lonza (Walkersville, MD, USA). Chemicals were dissolved in either appropriate media solution or dimethyl sulfoxide (DMSO) at the required working dilution. All chemicals were handled in accordance with the supplier’s recommendations. Anti-GRP78, p-EIF2a, EIF2a, ATF4, CHOP, LC3, bcl-2, bax, caspase-3 and β-actin antibodies were obtained from Cell Signaling Technology (Beverly, MA, USA). Insulin ELISA kits were purchased from Mercodia AB (Uppsala, Sweden).

### Cell culture and treatments

INS-1 cells were grown in RPMI 1640 medium supplemented with 10% FBS, 100 IU/mL penicillin, 100 g/mL streptomycin, 10 mM HEPES, 1 mM sodium pyruvate, 2 mM L-glutamine and 50 μM 2-mercaptoethanol at 37 °C in a humidified atmosphere containing 95% O_2_ and 5% CO_2_. βTC-6 cells were grown in DMEM containing 15% FBS, 100 IU/mL penicillin and 100 g/mL streptomycin in the same environment. Cholesterol was dissolved in the culture medium described above at a concentration of 10 mM and diluted to achieve a final working concentration of 5 mM. Our previous study indicated that cholesterol treatment at a concentration of 5 mM for 6 h induced cell apoptosis in pancreatic β-cells^27^; to further investigate the potential mechanism of lipotoxicity, we chose this concentration in our study. Cells were seeded in 6/12-well plates for 48 h before pretreatment with 1 mM 4-PBA for 24 h or 10 μg/mL E64d/pepstatin A for 1 h and then treated with vehicle (RPMI 1640 medium or DMEM) or 5 mM cholesterol for 6 h at 37 °C.

### Nuclear staining

Cells were fixed with 4% paraformaldehyde (pH 7.4) for 15 min at 4 °C and then treated with DAPI for 5 min at room temperature. After washing with PBS, stained cells were observed under an Olympus BX51 fluorescence microscope (Olympus Optical, Tokyo, Japan). Cells with intact and smooth nuclei were considered viable, whereas cells with nuclear shrinkage or fragmentation were considered apoptotic.

### Western blotting

Expression levels of target proteins were determined by western blot analysis. Cells were lysed on ice with RIPA lysis buffer containing a complete protease inhibitor mixture. Lysates were centrifuged at 12,000 × g at 4 °C for 15 min. The protein concentration in the supernatants was determined by the BCA method using bovine serum albumin (BSA) as the standard. Samples were separated by 10–12% SDS-PAGE, transferred to PVDF membranes that were incubated in 5% non-fat milk at room temperature for 1 h, and then incubated with the appropriate primary and secondary antibodies. Membranes were washed and proteins detected by enhanced chemiluminescence (ECL) using a LAS-4000 lumino-image analyzer (Fuji Film, Tokyo, Japan). Bands were digitally scanned and analyzed using ImageJ software (NIH Image, National Institutes of Health, Bethesda, MD, USA).

### Transmission electron microscopy (TEM)

Cells were fixed with 2.5% glutaraldehyde overnight at 4 °C and washed with cold PBS. After postfixing with fixative solution containing 1% osmium tetroxide for 1 h, cells were dehydrated with ascending grades of alcohol. Cells were then infiltrated and embedded in Spurr resin. Ultrathin sections were obtained using Leica EM UC7 (Leica, Wetzetlar, Germany) and stained with uranyl acetate and lead citrate. Cells were observed and photographed under a transmission electron microscope (Hitachi Model H-7650, Tokyo, Japan).

### Immunofluorescence

Cells were stained with 1 μM LysoTracker Red Lysosomal Probe in RPMI 1640 medium for 1.5 h at 37 °C. After washing with PBS, cells were fixed with 4% paraformaldehyde (pH 7.4) for 15 min at 4 °C, perforated with 0.5% Triton-100 for 15 min and blocked with 5% BSA for 1 h at room temperature. After incubation with the primary antibody anti-LC3 overnight at 4 °C followed by the secondary antibody, cells were treated with DAPI for 5 min and analyzed using an Olympus BX51 fluorescence microscope (Olympus Optical, Tokyo, Japan).

### Insulin secretion

Cells were washed with PBS and equilibrated in Kerbs-Ringer bicarbonate buffer (KRBB, pH 7.4) containing 140 mM NaCl, 1.5 mM CaCl_2_, 0.5 mM KH_2_PO_4_, 3.6 mM KCl, 0.5 mM MgSO_4_, 2 mM NaHCO_3_, 10 mM HEPES, and 0.1% BSA at 37 °C for 30 min. The buffer was removed and replaced with fresh KRBB containing 0 or 25 mM glucose for 1 h. Supernatants were collected, and the insulin concentration was measured using an insulin ELISA kit after appropriate dilution. Total protein was extracted with RIPA lysis buffer supplemented with 1 mM phenylmethyl sulfonylfluoride, and the protein concentration was determined using a BCA protein assay kit. The levels of insulin secretion were normalized against the respective protein content. Insulin secretion following stimulation with 0 and 25 mM glucose was defined as basal insulin secretion and glucose-stimulated insulin secretion, respectively.

### Statistical analysis

All data were calculated as the means ± SD, and checked using the Kolmogorov-Smirnov (KS) test before further analysis. Statistical significance between two datasets was assessed using the Student’s *t* test. Multiple groups were compared using a one-way ANOVA followed by Tukey’s multiple comparison testing. A *P* value of <0.05 was considered statistically significant. All statistical tests were performed using GraphPad Prism Version 6.0 (GraphPad Prism Software, Inc. CA, USA).

## Additional Information

**How to cite this article:** Kong, F.-J. *et al*. The endoplasmic reticulum stress/autophagy pathway is involved in cholesterol-induced pancreatic β-cell injury. *Sci. Rep.* **7**, 44746; doi: 10.1038/srep44746 (2017).

**Publisher's note:** Springer Nature remains neutral with regard to jurisdictional claims in published maps and institutional affiliations.
